# Two Great Men Gone

**Published:** 1915-11

**Authors:** 


### Two Great Men Gone.

The papers have been so filled with news of turbulence and bloodshed that they have given little space, comparatively, to the passing of two men who have contributed much to the preservation of the human race.

No one can tell how many thousands of lives, nor how many millions of dollars have been saved by the discovery that yellow fever was conveyed by a mosquito, the female stegomyia. But men now living who served in the Spanish-American war know what a terrible scourge that dread disease was to the regions in Central and South America, where it was endemic, and how this country, to which it was brought from those regions, dreaded it in its epidemic form. For 200 years before this war this country was subject to such epidemics, producing death by the thousands, business stagnation, poverty, untold suffering. In 1878 over 13,000 people in the Mississippi valley lost their lives from such an epidemic, and the loss of wealth was estimated at more than $100,-000,000.

Last month at Havana there died, full of years and honors, Dr. Carlos Finlay, who from the year 1881 until the Americans undertook the job of cleaning Havana, and among other things appointed a board of army medical officers to investigate yellow fever, had been investigating, thinking, and writing about the relation of the mosquito to yellow fever. He was convinced that the stegomyia conveyed yellow fever, and though it required the Reed board to establish his theory by experimentation, Dr. Reed of this board says: "To Dr. Carlos Finlay of Havana must be given full credit for the theory of the propagation of yellow fever by means of the mosquito, which he proposed in a paper read before the Royal Academy in that city at its session on the 14th day of August, 1881."

This discovery made possible sanitation in Havana which was so efficacious that since 1901 there has been only one case of yellow fever in that city, where yellow fever had existed continuously, been endemic, from 1762 until 1901, so that Dr. Gorgas says that there was probably not a single day when Havana did not have a case of yellow fever.

What the discovery meant to the building of the Panama canal all the world knows.

Surely Dr. Finlay deserves more signal honors than the greatest of generals leading his destroying armies into the field.

In Germany, at about the same time that Dr. Finlay died, Dr. Paul Ehrlich, the discoverer of salvarsan, the remedy for syphilis, died. His work was not to extend Germany's dominion, nor to bring glory to its destroying army. He served all mankind, and so won for Germany greater glory than has any of her war lords. —*Chamberlain's.*

